# Complete Genome Sequence of Escherichia coli Phage Paul

**DOI:** 10.1128/MRA.01093-19

**Published:** 2019-10-10

**Authors:** Ashley Holt, Robert Saldana, Russell Moreland, Jason J. Gill, Mei Liu, Jolene Ramsey

**Affiliations:** a Center for Phage Technology, Texas A&M University, College Station, Texas, USA; Queens College

## Abstract

Escherichia coli is both a commensal and a pathogen in humans and other animals. Here, we describe the isolation of E. coli strain 4s bacteriophage Paul. The complete 79,429-bp genome was annotated and demonstrates similarity with phieco32viruses, as does its prolate podophage morphology.

## ANNOUNCEMENT

Escherichia coli is a commensal bacterial inhabitant of the intestines, with pathogenic groups that cause human disease (1). E. coli strain 4s is a commensal isolate collected from horse feces and has an O-antigen component of the lipopolysaccharide known to affect susceptibility to phage (2). Here, we present the complete, annotated genome sequence of the E. coli 4s prolate podophage Paul.

Bacteriophage Paul was isolated from a filtered (0.2-μm-pore-size) water sample collected at Wolf Pen Creek in College Station, TX. The phage was propagated on E. coli 4s aerobically at 37°C in Luria-Bertani broth (BD Difco) using the soft-agar overlay methods described by Adams (3). DNA was purified with the modified Promega Wizard DNA clean-up system shotgun library preparation protocol (4), prepared as Illumina TruSeq Nano low-throughput libraries, and sequenced on an Illumina MiSeq platform with paired-end 250-bp reads using V2 500-cycle chemistry. The 2,820,474 reads in the phage index were quality controlled using FastQC (https://www.bioinformatics.babraham.ac.uk/projects/fastqc/). Sequence reads were then trimmed using the FASTX-Toolkit v0.0.14 (http://hannonlab.cshl.edu/fastx_toolkit/). The genome was assembled into a single contig with 1,429.4-fold coverage using SPAdes v3.5.0, with default parameters, and was confirmed to be complete by Sanger sequencing of a PCR product amplified off the raw contig ends (forward primer, 5′-CGTCGGCAATATCGTCTACTTT-3′, and reverse primer, 5′-AACAGCCTTACAATCCCTTACTG-3′) (5). Structural annotations were performed with GLIMMER v3.0 and MetaGeneAnnotator v1.0, and tRNA sequences were detected with ARAGORN v2.36 (6–8). Rho-independent termination sites were annotated using TransTermHP v2.09 (9). Gene functions were predicted using InterProScan v5.33-72, BLAST v2.2.31, and TMHMM v2.0, with default settings (10–12). BLAST searches were executed against the NCBI nonredundant and UniProtKB Swiss-Prot/TrEMBL databases with a 0.001 maximum expectation value (13). Structural predictions were done with the HHSuite v3.0 tool HHpred (multiple-sequence alignment [MSA] generation with HHblits using the ummiclus30_2018_08 database and modeling with the PDB_mmCIF70 database) (14). Genome-wide DNA sequence similarity was calculated by progressiveMauve v2.4.0, with default parameters (15). The annotation tools were accessed in the Galaxy and Web Apollo tools hosted by the Center for Phage Technology (https://cpt.tamu.edu/galaxy-pub) (16, 17) and run with default parameters (unless otherwise stated). The morphology of phage Paul was determined from samples negatively stained with 2% (wt/vol) uranyl acetate and viewed by transmission electron microscopy at the Texas A&M Microscopy and Imaging Center (18).

Paul is a 79,429-bp prolate podophage with 42.0% G+C content and 91.4% coding density. Structural annotations yielded 133 predicted protein-coding genes and a single tRNA gene. By BLASTp, Paul shares 113 proteins similar to those of enterobacteria phage phiEco32 (GenBank accession number EU330206), a 77-kb prolate podophage isolated against E. coli from cattle with acute mastitis (19). At the nucleotide level, Paul is most similar to other Phieco32virus members, including phage vB_EcoP_SU10 (82.24%, KM044272), phiEco32 (82.03%, EU330206), enterobacteria phage NJ01 (81.67%, JX867715), and Escherichia phage 172-1 (80.63%, KP308307). PhageTerm predicted 193-bp direct terminal repeats, and the assembled genome was reopened at the left terminal repeat boundary, syntenic with phiEco32 (20).

### Data availability.

The genome sequence and associated data for phage Paul were deposited under GenBank accession number MN045231, BioProject number PRJNA222858, SRA number SRR8892204, and BioSample number SAMN11411459.
